# Magnitude and factors associated with late antenatal care booking on first visit among pregnant women in public health centers in central zone of Tigray Region, Ethiopia: A cross sectional study

**DOI:** 10.1371/journal.pone.0207922

**Published:** 2018-12-05

**Authors:** Teklit Grum, Ermyas Brhane

**Affiliations:** 1 Department of Reproductive Health, College of Health Sciences, Aksum University, Aksum, Ethiopia; 2 Department of Human Nutrition, College of Health Sciences, Aksum University, Aksum, Ethiopia; Universitat Autonoma de Barcelona, SPAIN

## Abstract

**Background:**

Antenatal care (ANC) is a care given for pregnant women and is a good opportunity to deliver maternal health interventions. Even though pregnant women should start their first antenatal care within 12 weeks of gestational age, many pregnant women start their first ANC late. So, the aim of this study is to determine magnitude of late ANC booking at first visit and factors associated with it.

**Methods:**

Institutional based cross sectional study design was conducted in central zone of Tigray Region, Ethiopia from November 1/2017 to January 30/2018 among total of 632 pregnant women. Stratified multi stage cluster sampling method was used to select health centers and systematic random sampling technique was used during the selection of study units. Data were collected using interview administer questionnaire by face to face. The collected data were entered into EPI info-7. Later on, it was exported to STATA-14 for further analysis. Proportion was used to estimate the magnitude of late ANC booking. Bivariable and multivariable analysis were done to see factors associated with the magnitude of late ANC booking.

**Results:**

The magnitude of late ANC booking at first visit were 85.67% (95%, CI: 82.89, 88.45). Factors that were independently associated with the late ANC booking at first visit in multivariable analysis were; having home delivery in previous delivery (AOR = 2.2, 95%, CI: 1.1, 4.49), women who had no previous ANC follow up (AOR = 3.43, 95%, CI: 1.32, 8.92) and women with poor knowledge about the advantage and service availability of ANC (AOR = 3.9, 95%, CI: 1.83, 8.29).

**Conclusion:**

In summary, most of pregnant women were not started their first ANC at the recommended time. Home delivery and history of ANC in previous pregnancy as well as women with poor knowledge about ANC were associated with late ANC booking at first visit. Health workers should work on avoiding home delivery and increasing the knowledge of pregnant women on ANC may help on reducing late ANC booking at first visit.

## Introduction

### Background

Antenatal care is a type of care given for pregnant women and is considered as a key maternal service in improving a wide range of health outcomes for women and newborn. WHO ANC model recommends a minimum of eight ANC contacts, with the first contact scheduled to take place within the first trimester[1].

ANC creates a good opportunity to deliver interventions for improving maternal nutrition, providing health education, and encouraging skilled attendance at birth and use of facilities for emergency obstetric care[2, 3].

Implementing appropriate services and evidence-based practices during ANC at recommended time can improve maternal and fetal health[4, 5]. Adverse pregnancy outcomes can be minimized or avoided by provision of iron and folic acid supplementation which is better if provided during first trimester of pregnancy[6]. As the organogenesis of fetus mainly occurs during the first trimester, supplementation of essential elements have significant reduction on risk of having a baby with neural tube defects such as anencephaly and spinal bifida[1].

When women initiate ANC late, they have an increased risk of maternal and neonatal mortality[7, 8]. Many pregnant women in sub-Saharan Africa start their first antenatal care attendance late, mostly in the second and third trimester[3, 9]. Thus, late antenatal attendance makes difficult to implement effectively the routine ANC strategies that enhance maternal wellbeing and good prenatal outcomes [10–12].

Despite the fact that early antenatal care seeking is essential; little is known about factors with late booking for first ANC visit. Therefore, the aim of this study is to assess the magnitude and factors associated with late booking for first ANC.

## Methods and materials

### Study design

Institutional based cross sectional study design was conducted.

### Study period and area

The study was conducted in central zone of Tigray Region, Ethiopia from November 1/2017 to January 30/2018. The central zone is one of the seven zones found in Tigray region, Ethiopia which is 1050 KM far from the Addis Ababa (Capital city of Ethiopia) which encompasses nine district and three towns. Based on the 2007 census, this zone has a total population of 1,245,824, of whom 613,797 are men and 632,027 are women[13].

### Source population

All pregnant women who attend antenatal care in central zone of Tigray Region during the study period.

### Study population

All pregnant women who attend antenatal care in randomly selected health centers in central zone of Tigray Region during the study period.

### Inclusion criteria

Pregnant mothers who are attending 1^st^ ANC visit and above at the same health center. The timing of first ANC visit for the women who came for the second visit or above were taken from the longitudinal ANC register.

### Exclusion criteria

Women who attend first ANC at another facility but came to selected health center. These women may not be known their gestational age at their first visit and were excluded.

### Sample size determination

The sample size was estimated using single proportion formula using epinfo-7 from the study conducted in Adigrat town in which the proportion of women with late antenatal care booking were 51.8%[14]. Therefore, the total sample size was calculated with the assumption of 0.05 marginal error and 95% confidence interval. Based on these assumptions, the sample size was estimated as 383. After considering 1.5 design effect and adding 10% for non-respondents the final sample size was taken as 632.

### Sampling procedure

**Fig 1:**
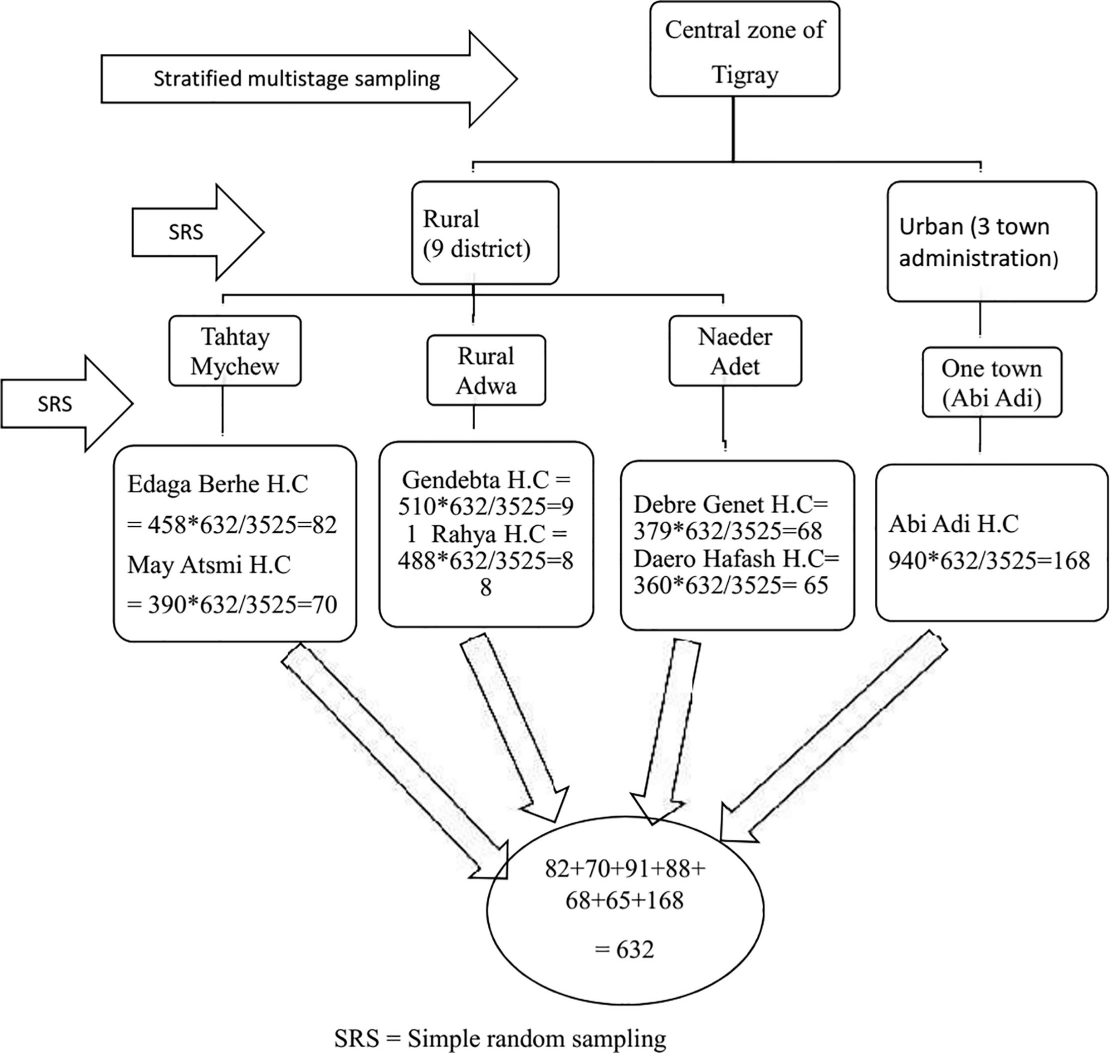
The schematic presentation of sampling procedures.

Stratified multi stage cluster sampling method was used during the selection of health centers. We stratified as rural and urban district. Out of nine rural district and three town administration, three rural district and one town administration was randomly selected. Then two health centers from each rural district and one health center from urban was selected randomly. By taking the quarter report ANC visit load in health centers, we allocated the required sample size using the proportional to sample size among the health centers. Systematic random sampling technique was used to select the study units (Fig 1).

### Data collection procedure and instrument

The data was collected using pretested structured questionnaire administered interview using face to face. Pregnant women’s knowledge on advantage and services availability of ANC were asked using 10 questions. The questionnaire was developed from different literatures. It was prepared in English and translated to Tigrigna (local language). Later on, it was translated back to English to check its consistency. The local language version of questionnaire was used to collect the data. Seven data collectors and two supervisors were hired during the data collection period.

### Data processing & analysis

After each questionnaire was checked for its completeness and consistency, data was coded and entered to EPI info-7 then exported to STATA-14 for further analysis. Descriptive analysis was used to describe the study variables using frequency, mean and percentage. Bivariable logistic regression analysis was made using OR and 95% CI to assess the association of independent variable with the outcome separately. Variables which were significant in the Bivariable logistic regression analysis at p-value <0.05 were included in to multivariable logistic regression analysis to see the association of independent variables with late antenatal care booking after controlling confounders. Multicollinearity was checked among the independent variables using variance inflation factor (VIF) and value above 10 was considered as determining the presence of multicollinearity. The necessary assumption of model fitness during logistic regression was checked using Hosmer and Lemeshow goodness of fit test statistics. The Independent variables that were significantly associated at p-value<0.05 with late ANC booking in multivariable logistic regression analysis were declared as a significantly associated. Measure of association were calculated using adjusted odds ratio (AOR) with 95% confidence interval.

### Data quality assurance

Data collectors along with the supervisors were trained for two days regarding to purpose of the study, data collection procedures, study tools and handling on collected data. During the data collection period, close supervision was made daily by the supervisors. Internal consistency were checked using Cronbach’s alpha.

### Ethical consideration

The study was approved by the Institutional Review Board of the College of Health Science Aksum University. Official letter was written to district health offices and to the health center directors. In addition, informed written consent was obtained from the respondent mothers after explaining the purpose of the data collection.

### Operational definition

Late ANC booking: Pregnant women were considered late ANC booking at first visit when they came to health facility after 12 weeks of gestational age[1].

Good Knowledge: Respondents were categorized as good knowledge on ANC if they scored above or equal to the mean knowledge score questions and Poor Knowledge when they scored below the mean knowledge score questions.

## Results

### Socio-demographic and economic characteristics of study subjects

The mean age of pregnant women were 27 with standard deviation (SD) of 7. Most of the study participants 533(86.81%), 589(95.93%) and 384(62.54%) were married/living together, orthodox religion and with farmer occupation respectively. Concerning to level of education, only 57(9.28%) and 51(8.31%) of the pregnant women and their husbands were with diploma or above respectively. Two hundred seventy nine (45.44%) of the pregnant women were with low wealth quintile. Majority of the study participants, 399(64.98%) were with less than 5 household size (Table 1).

**Table 1:** Socio-demographic and economic characteristics of pregnant women attending ANC in central zone of Tigray Region, Ethiopia in November 1/2017 to January 30/2018.

Variables	Category	Frequency	Percentage
Age Mean = 27 ± 6(SD)	<20	54	8.79
	20–34	471	76.71
	> = 35	89	14.50
Marital status	Unmarried	16	2.61
	Married/Living together	533	86.81
	Divorced	20	3.26
	Separated	45	7.33
Religion	Orthodox	589	95.93
	Muslim	25	4.07
Occupation	Housewife	82	13.36
	Farmer	384	62.54
	Merchant	87	14.17
	Employee	43	7
	Student	18	2.93
Woman’s level of education	No formal education	167	27.20
	Primary (1–8 grade)	268	43.65
	Secondary (9–12 grade)	122	19.87
	Diploma and above	57	9.28
Husband’s level of education	No formal education	166	27.04
	Primary (1–8 grade)	273	44.46
	Secondary (9–12 grade)	124	20.20
	Diploma and above	51	8.31
Wealth quintile	Low	279	45.44
	Medium	172	28.01
	Rich	163	26.55
Household size	< 5	399	64.98
	≥5	215	35.02

Among pregnant women who had history of pregnancy before were asked for their ANC experience and 334 (24.6%) were reported that they had previous ANC follow up. Regarding to the current pregnancy, 485(78.99%) of the study participant’s pregnancy were planned and 129(21.1%) were not planned. Seventy seven (82.93%) of the pregnant women had no history of abortion. Besides, 171(27.85%) of the participants were nulliparous. The magnitude of late ANC booking at first visit were 85.67% (95%, CI: 82.89, 88.45). Out of women who had history of giving birth, 137(30.93%) were reported as home delivery and only 36(8.13%) had history of still birth (Table 2).

**Table 2:** Obstetric history of pregnant women attending ANC in central zone of Tigray Region, Ethiopia in November 1/2017 to January 30/2018.

Variables	Category	Frequency	Percentage
Previous ANC	Yes	334	75.4
	No	109	24.6
Type of pregnancy	Planned	485	78.99
	Unplanned	129	21.01
History of abortion	Yes	77	17.07
	No	374	82.93
Parity	Nulliparous	171	27.85
	Multiparous	443	72.15
Timing of ANC at first visit	≤12 weeks of G.A	88	14.33
	>12 weeks of G.A	526	85.67
Place of previous delivery	Home	137	30.93
	Health facility	306	69.07
Birth interval	<3 years	171	38.60
	≥3 years	272	61.40
History of still birth	Yes	36	8.13
	No	407	91.87

Socio-demographic factors which were not significant association in Bivariable analysis were; age of pregnant women, marital status, occupation of women, husband’s level of education, wealth quintile and household size. Besides, obstetric factors; pregnancy planning, history of abortion, parity, birth interval and history of still birth has no shown any association with outcome variable in the Bivariable analysis. Women’s level of education were significant association with late ANC booking at first visit in Bivariable analysis but it remains insignificant in multivariable analysis.

Place of previous delivery was found that significant association with late ANC booking at first visit in multivariable analysis. Women who gave birth at home in their previous delivery were 2.2 times higher to be late booking than women who gave their previous delivery at health facility (AOR = 2.2, 95%, CI: 1.1, 4.49). Similarly women who had no history of ANC in previous pregnancy were more likely to be late booking (AOR = 3.43, 95%, CI: 1.32, 8.92). Knowledge on ANC were also significantly associated with late ANC booking at first visit. Women those who had poor knowledge about ANC were 4.8 times higher to be late on ANC booking at first visit (AOR = 3.9, 95%, CI: 1.83, 8.29) (Table 3).

**Table 3:** Bivariable and multivariable analysis on factors associated with late ANC booking at first visit.

Variables	Category	Late ANC booking at first visit		COR (95%, CI)	AOR (95%, CI)
		Yes (%)	No (%)		
Woman’s level of education	No formal education	145(27.57%)	22(25%)	0.47(0.22, 0.98) *	0.41(0.14, 1.15)
	Primary (1–8 grade)	228(43.35%)	40(45.45%)	0.54(0.27,1.08)	0.46(0.17, 1.25)
	Secondary (9–12 grade)	110(20.91%)	12(13.64%)	0.47(0.22, 1)	0.24(0.13, 1.44)
	Diploma and above	43(8.17)	14(15.91%)	1	1
Place of previous delivery	Home	127(33.07%)	10(16.95%)	2.42(1.2, 4.94) *	2.2(1.1, 4.49) *
	Health facility	257(66.93%)	49(83.05%)	1	1
Previous ANC	Yes	280(72.92%)	54(91.53%)	1	1
	No	104(27.08%)	5(8.47)	4.01(1.56, 10.3) *	3.43(1.32, 8.92) *
Knowledge on ANC	Good knowledge	287(54.56%)	75(85.23%)	1	1
	Poor knowledge	239(45.44%)	13(14.77%)	4.8(2.6, 8.87)*	3.9 (1.83, 8.29) *

*→p-value <0.05, COR: crude Odds Ratio, AOR: Adjusted Odds Ratio

## Discussion

We conducted a study on magnitude of ANC booking at first visit and factors that are associated for late ANC booking. WHO ANC model recommends pregnant women should attend ANC with the first contact scheduled to take place within the first trimester. However more pregnant women are not started their first ANC based on the WHO recommendation.

In this study, the magnitude of late ANC booking at first visit were found that 85.67% (95%, CI: 82.89, 88.45). This magnitude of late booking is slightly higher than studies conducted in East Wollega 81.5%[15], Gondar town 74.6%[16] and Kembata Tembaro zone 68.8%[17]. This implies that significant proportion of pregnant women are not started their first ANC visit at recommended time of gestational age. In Ethiopia, ANC coverage is raised from 34% in 2011[18] to 62% in 2016[2]. This indicate the government is committed towards increasing the coverage but focus was not given to the timing of ANC. So, raising the ANC coverage only may not be adequate to the pregnant women rather they should utilize the service in the optimal time of gestational age.

The first visit offers an opportunity to establish baseline information on the general wellbeing of the mother and the pregnancy. Taking iron and folic acid supplementation in pregnancy early in first gestational age benefits the pregnant women and fetus[7]. But late ANC booking at first visit indicates most pregnant mothers are not benefited from the services at recommended time.

In this study place of previous delivery was found that significantly associated with late ANC booking at first visit. Besides, Women who had no history of ANC in previous pregnancy were more likely to be late booking comparing to those who had ANC follow up in their previous pregnancy. Similar finding was reported in southern Ethiopia[17]. This could be due to pregnant women’s benefit from previous ANC exposure, linkages and familiarities with the health facility.

In the current study knowledge on ANC were also significantly associated with late ANC booking at first visit. Women those who had poor knowledge about advantage of ANC and services availability were more likely to be late on ANC booking at first visit. This finding is similar with a systematic review and meta-analysis study conducted on delayed initiation of ANC[8]. This implies if women were informed about the advantage and service availability of ANC, they can increase the service utilization at recommended time.

## Conclusion and recommendation

Most of the pregnant women were not started their first ANC visit on the recommended gestational age of pregnancy. The Ethiopian government has worked a lot on the ANC coverage improvement but the appropriate timing for first ANC has not yet evolved. Which shows pregnant women are not received base line services and other health interventions at early pregnancy. History of home delivery and no ANC in previous pregnancy were independently associated with the late ANC booking at first visit. Having poor knowledge on advantage and service availability of ANC were also associated with the outcome variable. Health workers should work on avoiding home delivery and increasing the knowledge on advantage of ANC for pregnant women.

## Supporting information

S1 Dataset (DTA)

## Abbreviations

ANC: Antenatal Care
AOR: Adjusted Odds Ratio
CI: Confidence Interval
COR: Crude Odds Ratio
SD: Standard Deviation
WHO: World Health Organization
